# Co-Design of a Sexuality Support Training Program for Practitioners Supporting People With Mental Illness (RIKASEKU-SAPOPRO): Protocol for a Randomized Controlled Trial

**DOI:** 10.2196/86419

**Published:** 2026-05-19

**Authors:** Masako Kageyama, Momoko Kusaka, Keiko Yokoyama, Taizo Shiraishi, Kazutaka Nishio, Shintaro Noma, Toshifumi Nemoto, Sohei Yogoh, Kayo Ichihashi, Emiko Nishioka, Nobuhiro Hara, Sachiko Takahashi

**Affiliations:** 1 Division of Health Sciences, Graduate School of Medicine, The University of Osaka, Yamadaoka 1-7, Suita, Osaka, 565-0871, Japan, 81 6-6879-2550, 81 6-6879-2550; 2 Department of Nursing, Faculty of Medical Sciences, Shonan University of Medical Sciences, Yokohama, Kanagawa, Japan; 3 Graduate School of Nursing, Yokohama Soei University, Yokohama, Kanagawa, Japan; 4 Q-ACT, Kitakyusyu, Fukuoka, Japan; 5 Department of Medical Innovation, The University of Osaka Hospital, Suita, Osaka, Japan; 6 AIRIKI Peer-Led Learning Program, Suita, Osaka, Japan; 7 Department of Neuropsychiatry, University of Tokyo Hospital, Tokyo, Japan; 8 Department of Maternal Nursing, Faculty of Health Science and Nursing, Juntendo University, Mishima, Shizuoka, Japan; 9 Town Counseling Center ‘Holon’, Fukuoka, Japan; 10 Center for Medical Education and Career Development and Community Health Science, Saitama Medical University, Moroyama, Saitama, Japan

**Keywords:** protocol, intervention, sexuality, mental illness, sexuality education, mental health professionals

## Abstract

**Background:**

Holistic health care includes sexuality. For people with mental illness, sexuality is an essential element of recovery. However, sexuality is often overlooked in mental health settings in Japan and elsewhere. Therefore, practitioners need to recognize sexuality as a legitimate area of care and acquire the skills and attitude**s** required to provide appropriate support. To address this gap, we developed a training program for practitioners through coproduction among peers with mental illness, researchers, and practitioners.

**Objective:**

This randomized controlled trial (RCT) in Japan compares an intervention group that participates in the program with a control group that does not and evaluates the program’s effectiveness in improving practitioners’ knowledge, attitudes, and confidence in providing sexuality-related support to clients with mental illnesses.

**Methods:**

The study participants are qualified practitioners aged 18 years or older who provide community-based support to people with mental illness. This 4-hour program combines comprehensive sexuality education with content specific to mental illness and deepens participants’ knowledge and skills through textbooks and case studies. The program is delivered in small groups of up to 8 participants by 2 facilitators: a practitioner and a peer with mental illness. Peer facilitators have experience facilitating sexuality-related group programs. The study design is a 2-arm parallel group RCT. After a baseline assessment, participants will be randomly assigned to an intervention or control group. Participants in the intervention group will complete an online questionnaire at baseline (T1), immediately after the program (T2), and 1 month after the program (T3). Participants in the control group will not receive the program during the study period and will complete the same 3 assessments (T1 to T3). Outcomes focus on participant competence rather than client condition. The primary outcome is confidence in providing sexuality-related support, measured using the self-assessment scale (lack of quality for sexuality education). Secondary outcomes include attitudes regarding sexuality (Attitudes Toward Sexuality Scale), knowledge of sexuality education, and knowledge of preconception care for people with mental illness. The target sample size is 76.

**Results:**

Funding was awarded in April 2023. Recruitment began in June 2025, and as of March 12, 2026, 57 participants had enrolled, 46 of whom had completed the final assessment. Analysis has not yet been conducted, and final data collection is anticipated by December 2026.

**Conclusions:**

To our knowledge, this will be the first RCT on a sexuality support training program for mental health practitioners. If effective, the program may help practitioners provide better support to people with mental illness and, in the long term, contribute to their recovery.

## Introduction

### Background

When health care practitioners provide care, they emphasize a holistic approach [1], and sexuality is an integral part of that care [2]. The World Health Organization (WHO) [3] defines sexuality as “a central aspect of being human throughout life is sexuality, which encompasses sex, gender identities and roles, sexual orientation, eroticism, pleasure, intimacy, and reproduction.” Literature reviews have revealed that health care practitioners receive limited education on sexuality and would benefit from additional training [4,5].

The guidelines for comprehensive sexuality education, issued by the United Nations Educational, Scientific and Cultural Organization (UNESCO) in its *International Technical Guidance on Sexuality Education: An Evidence-Informed Approach* [6,7], have become the global standard. Comprehensive sexuality education has been implemented in many countries, primarily for adolescents [8-10].

In Japan, sexuality has long been a taboo topic [11]. After the first patient with AIDS was identified in 1985, an AIDS panic ensued, and sexuality education became widespread [12]. In 2002, a politician claimed that sexuality education was encouraging sexual activity, and sexuality education then declined sharply [12]. Sexuality education has lagged behind international standards for many years [13,14]. Only recently have school teachers worked on comprehensive sexuality education, including the development of teaching materials that reflect both the guidelines and the local cultural and social context [15]. Regarding sexual minorities, homosexuality has historically been regarded as an abnormal sexual desire. However, the term LGBT (lesbian, gay, bisexual, transgender/transsexual) has been widely used since the early 2010s, and public awareness has increased [16]. Nevertheless, a nationwide survey revealed that 11.3% of the general population did not understand the meanings of the questions about sexual minorities [17], indicating that understanding remains insufficient. Furthermore, 14% of people reported having negative feelings toward homosexual colleagues [17], indicating that acceptance of sexual minorities is still limited. Thus, in Japan, sexuality has been considered taboo and has not been adequately taught, and prejudice against sexual minorities still persists, leading to resistance to discussing sexuality [18].

Illness can affect sexuality, including in the context of mental illnesses [19,20]. Mental illness is a global public health challenge with a lifetime prevalence of 12% to 47.4% [21], with approximately half of individuals developing the illness by the age of 14 years [22]. Review studies on sexuality among people with various mental illnesses have shown high rates of unintended pregnancy, domestic violence, sexual dysfunction due to medication side effects, and divorce [19,23,24]. Furthermore, despite being sexually active, people with mental illness may experience psychological suffering, including sexual stigma, low sexual confidence, and unmet sexual desires [19,20,25,26].

However, sexuality is still largely ignored in mental health settings [27]. Mental health practitioners may avoid the topic because they see it as outside their scope of practice, give it low priority, or feel uncomfortable discussing it; similar barriers have been reported among health care practitioners more broadly [28,29]. Additional barriers in mental health care include concerns about worsening a client’s mental health condition and the view that sexuality is not part of recovery [30-33]. Recovery is now a central concept in mental health services and a key goal of rehabilitation [34], and romantic loneliness is associated with poor recovery [35]. Intimate relationships may therefore form part of recovery, and sexuality may be an important barrier to address [20]. In this study, sexuality is defined as an important part of building intimate relationships and as an essential element of recovery for people with mental illness.

For people with mental illness, 1 major problem is that practitioners rarely address sexuality [27]. In a survey of people with mental illness in Japan, 59% reported having no one to consult about sexuality, and among those who did, most turned to psychiatrists, friends, or family members, whereas only a few consulted psychologists, nurses, or social workers [36]. Health care practitioners often lack sufficient knowledge and appropriate attitudes regarding sexuality [18]. Mental health practitioners, therefore, need the knowledge, attitudes, and confidence required to provide counseling and comprehensive sexuality education as part of recovery-oriented care for people with mental illness.

Previous international studies have examined these barriers to addressing sexuality in mental health care [37]. These studies suggest that mental health practitioners lack knowledge and skills related to sexuality support [31] and require training to overcome these barriers [30]. However, there are few studies on the development of training about sexuality for people with mental illness, with only a small number of interventions focusing on training for mental health nurses [38,39], mainly for hospitalized patients rather than clients living in the community. Psychiatric hospitals are settings focused on crisis management, where sexuality support is reported to be less of a priority [40]. A systematic review also reports implicit norms that view inpatient sexual behavior as risky [41]. Institutions pose special challenges with patient sexuality, including physical space [27]. For these reasons, sexuality support in inpatient settings may differ substantially from sexuality support in community settings.

In Japan, policies have shifted from inpatient treatment to community care, and mental health practitioners increasingly support people with mental illness in community settings [42]. Community-based mental health practitioners include a wide range of professionals, such as nurses, social workers, occupational therapists, and others [43]. Because support related to sexuality is especially relevant to community settings [44], it is important that these practitioners are able to provide effective support. To our knowledge, however, no training program in Japan has been reported for mental health practitioners who support the sexuality of people with mental illness.

Against this background, we developed a support training program (RIKASEKU-SAPOPRO) through coproduction among peers with mental illness, researchers, and mental health practitioners. The program was based on the *International Technical Guidance on Sexuality Education: An Evidence-Informed Approach* [7] and was designed to enable mental health practitioners to support the sexuality of people with mental illness living in the community. The outcome variables—knowledge, attitudes, and confidence in providing sexuality-related support—were selected based on an intervention study of a sexuality support training program for hospital mental health nurses [39]. This study uses a randomized controlled trial (RCT) to compare an intervention group that participates in the program with a control group that does not.

### Objectives

This study evaluates the effectiveness of a sexuality support training program by examining whether practitioners gain knowledge, improve attitudes, and build confidence in providing sexuality-related support to their clients with mental illnesses.

## Methods

### Study Design

The study design is an RCT with a 2-arm parallel group comparison. Participants are randomly assigned to either the intervention or control group after the baseline assessment. Participants in the intervention group complete an online questionnaire at 3 points in time: baseline (T1), immediately after the program (T2), and 1 month after the program (T3). The control group receives no program during the study period and is also assessed 3 times (T1-T3). To ensure that all participants are given an opportunity to take part in the program, the control group is offered the program after the final assessment (T3) has been completed. This protocol was developed and is reported in accordance with the Standard Protocol Items: Recommendations for Interventional Trials (SPIRIT) 2013 checklist (Checklist 1).

### Study Setting

This study was conducted in Japan. Participants were recruited through a dedicated research website and through information distributed to community-based institutions and networks that support people with mental illness.

### Study Participants

The study participants are qualified practitioners aged 18 years or older who provide community-based support to people with mental illness. There are no restrictions on profession; eligible practitioners include physicians, nurses, social workers, psychologists, occupational therapists, and others. The certifying body of professional qualifications, such as a national or municipal authority, is not used as an eligibility criterion. Since the 21st century, Japan has experienced increased immigration, including labor immigration [45]. For this reason, no restrictions are placed on ethnicity, and ethnicity is collected as a demographic characteristic. The exclusion criteria are the inability to participate in the online survey or Zoom video conferencing and difficulty participating in group discussions in Japanese.

### Intervention Program

#### Program Coproduction Process

RIKASEKU-SAPOPRO is a sexuality support training program aimed at recovery. Although mental health services have traditionally been developed by researchers and health practitioners, the concept of coproduction with service users has recently been identified as central to the provision of effective recovery-oriented services [34]. This program has been developed through coproduction among 5 peers with mental illness (out of these 5 peers, 2 of them were qualified social workers) and 7 researchers and/or practitioners (4 nurses, 1 psychiatrist, 1 obstetrician-gynecologist, and 1 social worker). Among them, 2 are experts in sexuality education, and 1 is involved in awareness-raising activities as a sexual minority. We have already cocreated a program about intimate and romantic relationships with peers with mental illness [46], in which we worked with about 25 peer facilitators. We recruited peers who were interested in this research project to establish a research team and develop the current program.

From the initial idea of program development to program design, selection of evaluation criteria, and implementation, researchers, practitioners, and peers were equally involved, and all opinions were respected. To support equal participation, we adopted a nonhierarchical form of address and held social gatherings to build trust. The research team worked together for 2 years before the intervention started. During this period, a questionnaire survey of 300 people with mental illnesses [36], interview studies with 23 women with mental illness [47], and interviews with 26 practitioners were conducted, and the findings were reflected in the program textbook. All aspects, including the program’s structure, textbook materials, the style of facilitation in peer-practitioner pairings, and the defined outcome variables, underwent discussion and were finalized by this team. A total of 3 pilot programs were conducted and reviewed.

#### Program Overview and Development Process

Table 1 provides an overview of the program. The 4-hour program combines comprehensive sexuality education with content specific to mental illness, deepens knowledge through textbooks, and develops support skills through case studies. The program is conducted in small groups of up to 8 participants, with 2 facilitators: a health care practitioner and a peer with mental illness. The entire program is based on the *International Technical Guidance on Sexuality Education: An Evidence-Informed Approach* [7]. We use the sexuality education textbook “Mana Book” (level 4), which is based on the *International Technical Guidance on Sexuality Education: An Evidence-Informed Approach* and was first developed in Japan for individuals aged 15 to 18 years.

**Table 1.** Components of the RIKASEKU-SAPOPRO program^a^.

Modules and time duration (min)		Lesson	Purposes	Contents
Module 1—introduction key concepts: (1) “Relationships” and (2) “Values, Rights, Culture, and Sexuality”				
7		Introduction	Creating a safe place	Program overview and review of ground rules
8		Self-introduction	Getting to know each other and feeling safe	Description of usual work, motivation for participation, and icebreaker
10		Relationships and values	Gaining knowledge and awareness	Family structure, interpersonal boundaries, and reactions to sexuality
10		Sexuality support	Gaining awareness	Skills for counseling and responding to questions about the characteristics of sexual topics, the background and nature of worries, and values
20		Case studies (2 cases)	Acquiring support skills	A case where the client believes they are in love and a case where a woman is being sexually exploited but is unaware.
5		Reflection	Summarize own thoughts	Participants share their awareness and impressions.
Module 2—key concepts: (3) “Understanding gender,” (6) “The human body and development,” and (7) “Sexuality and sexual behavior”				
10		Sexual diversity	Gaining knowledge and awareness	SOGIE^b^
10		Gender roles, sexual dysfunction, and sexual pleasure	Gaining knowledge and awareness	Gender-based attitudes, sexual dysfunction, sexual response, and sexual pleasure in people with mental illness
25		Case studies (2 cases)	Acquiring support skills	A case of a person who has discovered they are a sexual minority and a case of unfulfilled sexual desires
5		Reflection	Summarize own thoughts	Participants share their awareness and impressions.
Module 3—key concepts: 5 “Skills for health and well-being”; 4 “Violence and staying safe”				
10		Sexual independence and sexual consent	Gaining knowledge and awareness	What is sexual autonomy and consent, and how does the media influence it?
10		Sexual violence and sexual crimes	Gaining knowledge and awareness	Survivors of domestic violence and sexual crimes among people with mental illnesses; criminal law reform regarding sexual violence and sexual crimes.
25		Case studies (2 cases)	Acquiring support skills	A case where sexual consent was mistakenly obtained, and a case of economic exploitation using romantic feelings
5		Reflection	Summarize own thoughts	Participants share their insights and impressions.
Module 4—key concept: (8) “Sexual and reproductive health”				
7		Preconception care and pregnancy	Gaining knowledge and awareness	Preconception care for people with mental illnesses, pregnancy, and contraception
13		Whether to have a child and pregnancy	Gaining knowledge and awareness	Examples of people with mental illness worrying about whether to have children, pregnancy anxiety, unexpected pregnancies, and sexually transmitted diseases
13		Case study (1 case)	Acquiring support skills	The case of a woman suffering from mental illness and anxiety about raising her children
10		How to consult	Gaining knowledge and awareness	Skills to receive SOS calls and provide counseling for people with mental illnesses
7		Overall comments	Program completion	Participants share their overall thoughts on the program.

a Structure: combination of comprehensive sex education and content specific to mental illness, designed to deepen knowledge and support skills through textbooks and case studies. The program is conducted in small groups of 8 people or fewer, led by a practitioner and a peer, and spans 4 hours.

b SOGIE: sexual orientation, gender identity, and gender expression.

In addition, an original textbook was developed to address concerns specific to mental illness. All modules include a textbook section for knowledge confirmation and a scenario-based case study section, which is a major strength of this training format [4]. After confirming their knowledge through the textbook, participants share their impressions and gain new insights by listening to the opinions of others. During discussions of the case studies, participants exchange opinions on the types of support that can be provided for each case.

#### Program Facilitators

The program is facilitated by pairing a peer with mental illness and a practitioner. In this study, peer facilitators are expected to provide participants with perspectives that differ from those of practitioners because they are experts based on their own lived experience. In Japan, there are no formal qualifications for peer supporters, and no training is required to act as a peer supporter. To recruit experienced peers for this program, we selected individuals from among peer facilitators in an intimate relationship group program [46,48]. Practitioner facilitators were initially recruited from among those who had cooperated with interviews about sexuality in order to identify individuals who were interested in the topic. Subsequently, we recruited practitioners who had completed the final assessment of the study by asking whether they wished to serve as facilitators. All practitioner facilitators hold national qualifications, such as social worker or nurse. All facilitators received 6 hours of training for this program. As of March 12, 2026, 10 peer facilitators and 14 practitioner facilitators had completed the training. A facilitator manual was created to promote adherence to the intervention protocol by specifying in detail the sections of text to be read and the time allotted for each discussion. M Kageyama observes all the intervention sessions to ensure that they are implemented according to the manual.

### Random Allocation and Blinding

KN prepared an allocation list stratified by assigned sex at birth (female or male). In Japan, policy guidance has generally favored assigning practitioners of the same sex for care that service users may perceive as sensitive or embarrassing, partly to protect the rights and dignity of people with disabilities [49]. Practitioner sex may therefore influence the effectiveness of the program, as providing sexuality-related support to same-sex versus opposite-sex clients may affect the content and type of counseling offered. In addition, understanding of sexual diversity remains limited in some settings in Japan; even health care practitioners may encounter discrimination related to sexual minority status [36], and some individuals may be reluctant to disclose their gender identity even when distressed [50]. These sociocultural factors influenced the study design and informed the decision to avoid stratification based on gender identity. Randomization was therefore stratified by sex assigned at birth in order to reduce the privacy burden associated with collecting sensitive personal information.

The allocation is performed using REDCap (Research Electronic Data Capture; Vanderbilt University), a widely used web application for building and managing online surveys and databases. KN prepared the allocation list before enrollment began. REDCap locks the randomization model to ensure that it is not modified once a study becomes active. After M Kageyama confirms that participants have responded to the online survey (T1), M Kageyama enters the assigned sex at birth data into REDCap, and random allocation is performed automatically. M Kageyama communicates the allocation results displayed in REDCap to participants by email. Group assignment is unknown to researchers other than M Kageyama. M Kageyama is the sole individual with access to the test dataset, which is managed through REDCap. M Kageyama is responsible for extracting group information from the final dataset and subsequently providing the dataset to M Kusaka for statistical analysis.

### Sample Size

We plan to recruit 76 participants. A previous study [51] conducted a similar intervention; in that study, the average scale score of the primary outcome variable before the training was 23.39, and the average scores 1 week and 3 months later were both 18.56. In our study, we assume that the intervention group’s average score 1 month after the intervention will be 18.56, compared with 23.39 for the control group. Furthermore, the previous study [51] that examined the validity of the scale reported an average of 19.5 (SD 5.5). In our study, taking into account the intervention and the characteristics of the participants, the SD is assumed to be 6.5.

Under the above assumptions, if a 2-tailed *t* test is used with an α error of 5% on both sides and a power of 80%, the required sample size is 60 (n=30 for each group). After considering a dropout rate of 20%, the required sample size is 76. The study’s intervention is administered on a group-by-group basis; however, given the nature of the intervention, the expected small group effect allows for a sample size calculation method that presumes independence.

### Procedure

Procedures for recruitment, eligibility determination, and enrollment are presented in Textbox 1

Textbox 1. **Procedures for recruitment, eligibility determination, and enrollment.**

- Potential candidates are recruited through a dedicated internet home page and community-based institutions that support people with mental illnesses.
- People interested in participating in the study should email M Kageyama.
- M Kageyama sends a study description to potential candidates by email or by post.
- Potential candidates read the study description, and if they have questions about the study, they submit their inquiries via email or phone. If they understand the study description and agree to participate, they send a written consent form to M Kageyama by email or post.
- Once the consent forms are received, M Kageyama assesses participants’ eligibility via email. The author confirms that potential candidates meet the inclusion criteria, such as providing support to people with mental illness in the community, holding relevant qualifications, and being at least 18 years old, and also confirms that they do not meet the exclusion criteria, such as an inability to use Zoom conferencing or online surveys and an inability to participate in group discussions in Japanese. Those who meet the criteria are enrolled as study participants.
- M Kageyama emails those who have confirmed their eligibility. Study participants complete the baseline questionnaire (T1) using REDCap (Research Electronic Data Capture; Vanderbilt University).
- M Kageyama uses REDCap to randomly allocate the study participants who have completed the baseline questionnaires (T1).
- M Kageyama informs participants by email about their allocated group and the upcoming schedule.
- For intervention groups, the textbook and “Mana Book” (level 4) are mailed to participants 2 weeks before the start of the program, and they are asked to read them before attending.
- Participants in the intervention group participate in the program within 2 months of allocation and complete questionnaires immediately after the program (T2) and 1 month later (T3), whereas participants in the control group complete questionnaires 1 month (T2) and 2 months (T3) after allocation.
- After completing the final questionnaire (T3), participants in the control group may voluntarily attend the program.

### Assessment Plan

#### Demographic Variables

Demographic data collected for study participants include age, ethnicity, assigned sex at birth, occupation, workplace, provision of individual support, management position, years of practice, years supporting individuals with mental illness, experience in providing sexuality-related support, experience receiving sexuality education, and motivation for participation.

#### Outcome Variables

##### Confidence in Providing Sexuality-Related Support

The primary outcome is confidence in providing sexuality-related support, measured using the self-assessment scale of the lack of quality of sexuality education [51]. This scale was originally developed to self-assess the quality of sexuality education that parents provide to their children. In this study, we replace “child” with “client.” It is a 6-item, 1-factor scale that has been examined for validity and reliability [51]. The items are: “I don’t know how to talk to the client about sexuality,” “I don’t know what and how much I should teach the client about sexuality,” “I can’t find a way to give them knowledge about sexuality,” “I feel embarrassed to talk to the client about sexuality,” “My knowledge of sexuality is insufficient to provide sexuality education,” and “I don’t know what the client knows about sexuality.” Responses to the 5-point Likert scale range from 1 (“Not at all applicable”) to 5 (“Very applicable”). Total scores range from 6 to 30. Lower scores indicate higher confidence in providing sexuality-related support.

##### Attitudes Toward Sexuality

Attitudes toward sexuality are a secondary outcome measured by the Attitudes Toward Sexuality Scale [52]. A liberal attitude toward sexuality within the profession is a prerequisite for recognizing the need for care regarding sexuality and for maintaining the flexibility to respond to clients’ diverse human values and ways of life [53]. The scale comprises 37 items and 5 subscales measuring attitudes toward sexuality, and has been examined for validity and reliability [52]. The subscales are: “Attitudes Toward Sexual Minorities” (12 items), “Attitudes Toward Sexual Reproductive Bias” (8 items), “Attitudes Toward Sexual Desire and Heterosexuality Among Older Adults and Women” (9 items), “Attitudes Toward Sexual Violence” (4 items), and “Repressive Attitudes Toward Female Sexuality” (4 items). Each item is rated on a 5-point Likert scale, with responses ranging from 1 (“Very much agree”) to 5 (“Very much disagree”). No overall score is obtained; instead, the sum of the scores for each of the 5 subscales is calculated. The higher the score, the more liberal the attitude toward sexuality; conversely, the lower the score, the more conservative the attitude.

### Knowledge of Sexuality Education

Knowledge of sexuality education is a secondary outcome. The items test knowledge about the content of the textbook “Mana Book” and include items such as “Outing is talking about things that pertain to your privacy that you have not shared before” [15]. The test consists of 30 items with “Correct,” “Wrong,” and “Don’t know” options. Scores are totaled, with 1 point awarded for each correct answer. Total scores range from 0 to 30.

### Knowledge of Preconception Care for People With Mental Illness

Knowledge regarding preconception care for people with mental illness is a secondary outcome. The items are based on perinatal clinical practice guidelines for women with mental illness published by academic societies [54]. The 5 items, such as “It is desirable for mental illness to be stable for at least 3 months before becoming pregnant” and “Most psychotropic medications are compatible with breastfeeding,” have been used in a survey [36]. Responses are “I knew” and “I didn’t know.” The scores (1 point for “I knew”) are totaled. Scores range from 0 to 5.

### Process Assessment

The process assessment is conducted only on the intervention group. It includes questions about satisfaction (multiple choice and free text), whether the program would be useful for future support (multiple choice and free text), whether they would recommend the program to other practitioners (multiple choice and free text), and what changes occurred (free text). How the support was reflected during the 1-month follow-up period will be shown through an analysis of the qualitative data obtained from “What changes occurred (free text).”

Demographic variables are collected during the baseline assessment (T1). Outcome variables are collected during the baseline assessment (T1), assessment 1 (T2), and assessment 2 (T3). A process assessment is conducted at T3 only for the intervention group. Table 2 shows the contents of the questionnaire.

**Table 2.** Content of the questionnaires.

Assessment	Demographic variables	Outcomes
T1 (baseline assessment)	✓	✓
T2 (following assessment 1)		✓
T3 (following assessment 2)		✓

### Study Hypotheses

The hypotheses of this study are (1) the intervention group will have higher confidence in providing sexuality-related support at T3 than the control group (hypothesis 1), (2) changes in confidence in providing sexuality-related support scores from T1 to T2 and T3 will be greater in the intervention group than in the control group (hypothesis 2), (3) scores on the Attitudes Toward Sexuality Scale at T3 will be higher in the intervention group than in the control group (hypothesis 3), (4) changes in the scores on the Attitudes Toward Sexuality Scale from T1 to T2 and T3 will be greater in the intervention group than in the control group (hypothesis 4), (5) scores for knowledge of sexuality education at T2 and T3 will be higher in the intervention group than in the control group (hypothesis 5), and (6) scores for knowledge regarding preconception care for people with mental illness at T2 and T3 will be higher in the intervention group than in the control group (hypothesis 6).

### Analysis Plan

#### Statistical Analysis

The primary analysis will be conducted on the full analysis set (FAS), with supplementary analyses performed using the per-protocol set. The FAS will include all enrolled participants except those who meet the following exclusion criteria: (1) no response at T1 and (2) not randomized. The per-protocol set will consist of participants in the FAS except those who meet the following exclusion criteria: (1) no response at T2 and/or T3, (2) assignment to the intervention group but attendance at fewer than half of the program sessions, and (3) withdrawal from the study during the intervention period.

Baseline data for continuous variables will be summarized as mean and SD or as median and IQR, whereas categorical variables will be summarized as frequencies and percentages.

For the main hypothesis 1, a Student 2-tailed *t* test will be conducted, and a 95% CI will be calculated. An analysis of covariance will be conducted with assigned sex at birth and baseline values included as covariates, and the difference in least squares means, along with its 95% CI, will be estimated.

For hypotheses 2 and 4, a mixed-effects model for repeated measures (MMRM) will be conducted with group, time points (T1, T2, and T3), and the interaction between group and time points as fixed effects, and participants as a random effect. The variance-covariance structure of the error terms in the MMRM will be tested in the following order: unstructured, compound symmetry, and auto-regression. The estimation results based on the first successful convergence of the specified structure will be adopted. MMRM estimation will be conducted using the restricted maximum likelihood method.

For hypothesis 3, the same analysis as that for the main hypothesis will be conducted.

For hypotheses 5 and 6, the Wilcoxon rank-sum test will be conducted at time points T2 and T3. Since these are secondary end points, no adjustments for multiplicity will be made.

All statistical analyses will be conducted using IBM SPSS (Statistical Package for the Social Sciences) Statistics (version 23).

#### Process Analysis

For categorical data, frequencies and percentages will be calculated for each group. Text data will be analyzed qualitatively. Specifically, M Kageyama will code the textual data by separating it into meaningful units. Categories will be created by increasing the level of abstraction based on the similarities and differences among codes. Once the categories are established, KY will classify the codes to assess consistency. M Kageyama and KY will address codes with mismatched classifications. If disagreement remains, M Kusaka will make the final decision.

### Monitoring

Data monitoring is performed by M Kageyama using REDCap. Because REDCap is used to collect questionnaire data, the date and time of questionnaire responses from T1 to T3 can be ascertained. The system is set up so that reminders to respond to the questionnaires are sent automatically 3 times at 2-day intervals. Participants are monitored to determine whether they complete the questionnaires within the 1-week time frame. If they do not respond by the deadline, they are contacted by email and then by phone if they do not respond to the email. If participants wish to withdraw from the study or do not respond to the questionnaires, M Kageyama will ascertain the reason by email or phone. The audit organization at the University of Osaka Hospital, which is independent of the researchers, conducts the audit at the end of the first case of intervention.

### Ethical Considerations

This study protocol has been evaluated and approved by the ethical review board at the University of Osaka Hospital (25040; 2025/6/18). Any future modifications to the protocol, should they arise, will be reviewed by the same ethical review board.

Written informed consent will be obtained from all participants. Study documents address ethical issues, including the study protocol, the voluntary nature of participation, the privacy policy, data management procedures, participant safety, and the absence of conflicts of interest. Study participants’ personal information is collected by M Kageyama via email, entered into a Microsoft Excel sheet, and password-protected for secure management before, during, and after the trial. After enrollment, if participants themselves declare their intention to withdraw, the intervention will be discontinued. At the end of the study, participants will receive a gift card worth JPY 3000 (approximately US $20) as a gesture of gratitude. The trial was registered with the UMIN Clinical Trials Registry (UMIN000058204; June 18, 2025).

### Dissemination

After completing this study, we will publish the results in an international peer-reviewed medical or nursing journal and present the results at national and international conferences. Information on publication will be announced on the research home page in accordance with the journal’s publication rules. Authorship will follow the guidelines of the International Committee of Medical Journal Editors [55].

## Results

**Figure 1.**
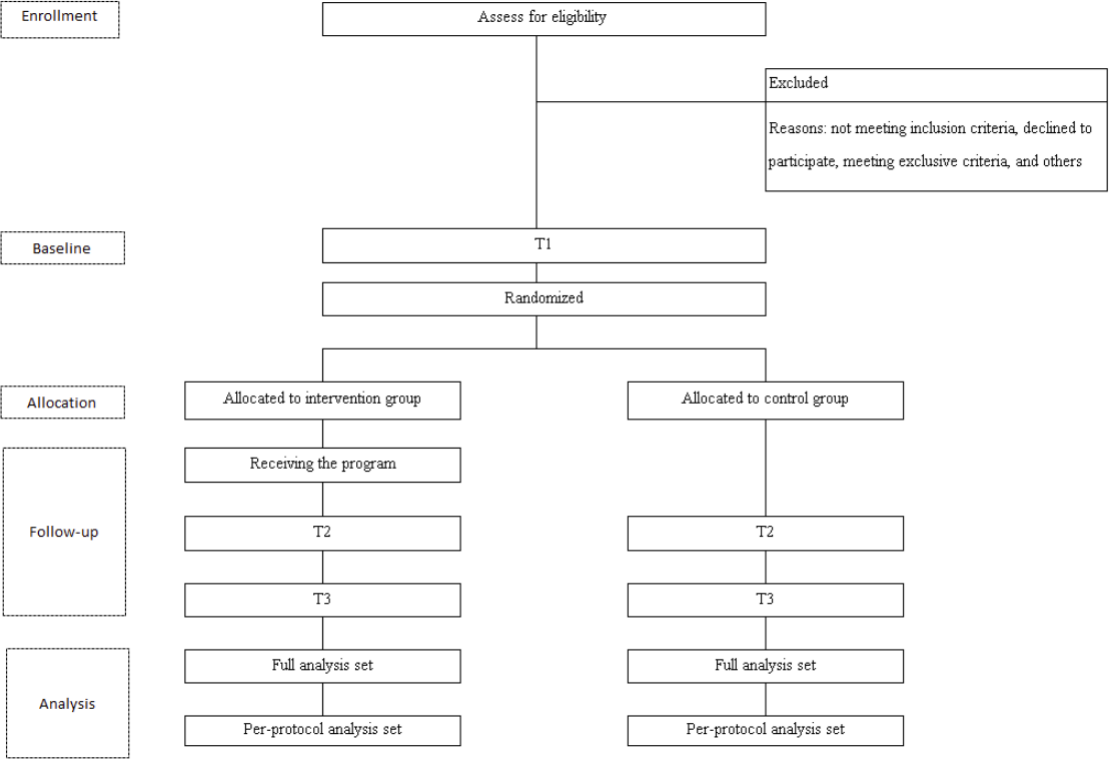
Flowchart of study participants.

Funding was awarded in April 2023. The flow diagram of the study participants is shown in Figure 1. Recruitment of participants began in June 2025. A total of 83 potential candidates expressed interest in participating in this study and were sent the study description. A total of 59 candidates submitted written consent forms. Out of the 59 candidates, 2 were not eligible because they were nurses working with inpatients at hospitals rather than clients living in the community. As of March 12, 2026, 57 participants had enrolled. The assigned sex at birth of the 57 participants was 44 females and 13 males. Their main qualifications were social worker (n=23), nurse (including midwife and public health nurse) (n=19), psychologist (n=7), doctor (n=5), counseling support specialist (n=2), and occupational therapist (n=1). The main workplaces supporting people with mental illness were counseling offices (n=30), rehabilitation facilities (n=9), home visiting stations (n=7), outpatient psychiatry settings (n=5), and others (n=6). Of these participants, 46 completed the final assessment. Regarding ethnicity, 1 participant was not Japanese. As of March 12, 2026, no participant had dropped out, and 2 participants completed the assessment late but still completed it. The audit organization conducted an audit at the end of the first intervention case, and no issues were identified. Analysis is not yet complete because it will be conducted after the final participant assessment has been completed, with the last data collection expected by December 2026.

## Discussion

This program is a training initiative for practitioners focused on providing sexuality support specific to mental illness. This study evaluates the program’s effectiveness by examining whether practitioners gain knowledge, improve attitudes, and build confidence in offering sexuality-related support to clients with mental illness.

### Principal Results

This training program is designed to enable practitioners to provide support regarding sexuality, with confidence in providing sexuality-related support as the primary outcome. A survey of people with mental illness in Japan revealed that they do not adequately use contraception or take measures against sexually transmitted diseases, and they lack the necessary knowledge before becoming pregnant [36]. Therefore, practitioners are required to provide sexuality education in a way that is tailored to each individual. The same survey also found that 18.3% of respondents with mental illness were sexual minorities [36], making it essential for practitioners who support people with mental illnesses to have a liberal attitude that accepts sexual diversity. If this study shows that practitioners in the intervention group have more knowledge about sexuality, a more liberal attitude, and greater confidence than those in the control group, it will provide evidence on the effectiveness of a coproduced sexuality support training program for practitioners working in community settings.

### Comparison With Prior Work

Two training programs for mental health practitioners regarding sexuality have been reported previously. One program is an 8-hour sexual health training program for mental health nurses in Taiwan [39], and the other is a 40-minute training program for mental health nurses in Australia [38]. The Taiwanese program covers a wide range of sexuality-related topics and includes not only lectures but also role-playing and discussions. This program improved knowledge, attitudes, and self-efficacy [39]. Compared with our program, it differs in that it targets mental health nurses working in hospitals, lasts 8 hours, and was evaluated using a quasi-experimental design. The Australian program focused more on counseling methods than on providing knowledge, and participants were evaluated through interviews to determine whether they were able to use the methods they learned in practice [38]. Compared with our program, it differs in that it targets mental health nurses, provides more advanced training, has a shorter training time, and uses interview-based rather than quantitative evaluation. Both programs are delivered by practitioners or researchers and are not facilitated by practitioner-peer facilitator pairs.

### Strengths and Limitations

The main strengths of this program are that it targets a variety of community-based practitioners, includes peer facilitators, and evaluates effectiveness through an RCT. In accordance with the emphasis on coproduction [34], implementing this collaborative program between practitioners and peers holds considerable importance. In Japan, training facilitators for practitioners are usually researchers or practitioners, and people with mental illness rarely serve as facilitators [56]. Therefore, for many participants, it will be their first experience attending a training session facilitated by a person with mental illness. We hope that by incorporating the perspectives of people with mental illness, practitioners will gain new insights into their experiences and adopt a more flexible attitude.

In Japan, there is no program for learning how to provide sexuality support specific to mental illness. If this program proves effective, mental health practitioners in Japan may enroll in it. If practitioners who complete the program are able to provide sexuality support to people with mental illness, it may help improve their long-term well-being.

Several limitations should also be noted. First, recruitment may not be easy. A review article [32] reported that mental health practitioners consider sexuality a taboo subject and not of high interest unless it is directly related to their work. Despite publishing articles in mental health magazines about the need for sexuality support and raising awareness to ensure that the topic does not remain taboo, we may need to recruit participants proactively. Second, the outcomes focus on practitioners’ knowledge, attitudes, and confidence rather than direct client-reported outcomes because participants may not have opportunities to provide sexuality support within the 1-month follow-up period. Third, randomization is stratified by assigned sex at birth rather than gender identity, reflecting privacy considerations in the Japanese context; this decision should be interpreted within that context.

### Future Directions

Given the substantial impact of culture and society on sexuality, it is imperative to develop health care practitioner training curricula tailored to each nation [7]. Continued program development should value coproduction with people with mental illness [34]. Future research may involve extending the follow-up period to design studies that more effectively measure the impact of the program in practice. Furthermore, future work should examine how coproduced programs can be implemented more widely and disseminated across community mental health settings.

## Supplementary material

10.2196/86419 Checklist 1 SPIRIT checklist.

## Abbreviations

FAS: full analysis set
LGBT: lesbian, gay, bisexual, transgender/transsexual
MMRM: mixed-effect model for repeated measures
RCT: randomized controlled trial
REDCap: Research Electronic Data Capture
SPIRIT: Standard Protocol Items: Recommendations for Interventional Trials
SPSS: Statistical Package for the Social Sciences
UNESCO: United Nations Educational, Scientific and Cultural Organization
WHO: World Health Organization
